# State-of-the-art pulsed field ablation for cardiac arrhythmias: ongoing evolution and future perspective

**DOI:** 10.1093/europace/euae134

**Published:** 2024-06-07

**Authors:** Kyoung-Ryul Julian Chun, Damijan Miklavčič, Konstantinos Vlachos, Stefano Bordignon, Daniel Scherr, Pierre Jais, Boris Schmidt

**Affiliations:** CCB Frankfurt, Med. Klinik III, Markuskrankenhaus, Wilhelm-Epstein Str. 4, 60431 Frankfurt, Germany; Klinik für Rhythmologie, UKSH, Ratzeburger Allee 160, 23538 Lübeck, Germany; Faculty of Electrical Engineering, Laboratory of Biocybernetics, University of Ljubljana, Trzaska cesta 25, SI-1000 Ljubljana, Slovenia; Site Hôpital Xavier Arnozan, Bordeaux University Hospital, University of Bordeaux, Avenue du Haut-Lévêque, –Pessac, France; CCB Frankfurt, Med. Klinik III, Markuskrankenhaus, Wilhelm-Epstein Str. 4, 60431 Frankfurt, Germany; Klinische Abteilung für Kardiologie, Medizinische Universität Graz, Auenbruggerplatz 15, 8036 Graz, Austria; Site Hôpital Xavier Arnozan, Bordeaux University Hospital, University of Bordeaux, Avenue du Haut-Lévêque, –Pessac, France; CCB Frankfurt, Med. Klinik III, Markuskrankenhaus, Wilhelm-Epstein Str. 4, 60431 Frankfurt, Germany

**Keywords:** Catheter ablation, Energy sources, Electroporation, Pulsed field

## Abstract

Pulsed field ablation (PFA) is an innovative approach in the field of cardiac electrophysiology aimed at treating cardiac arrhythmias. Unlike traditional catheter ablation energies, which use radiofrequency or cryothermal energy to create lesions in the heart, PFA utilizes pulsed electric fields to induce irreversible electroporation, leading to targeted tissue destruction. This state-of-the-art review summarizes biophysical principles and clinical applications of PFA, highlighting its potential advantages over conventional ablation methods. Clinical data of contemporary PFA devices are discussed, which combine predictable procedural outcomes and a reduced risk of thermal collateral damage. Overall, these technological developments have propelled the rapid evolution of contemporary PFA catheters, with future advancements potentially impacting patient care.

## Graphical Abstract

**Graphical Abstract:**
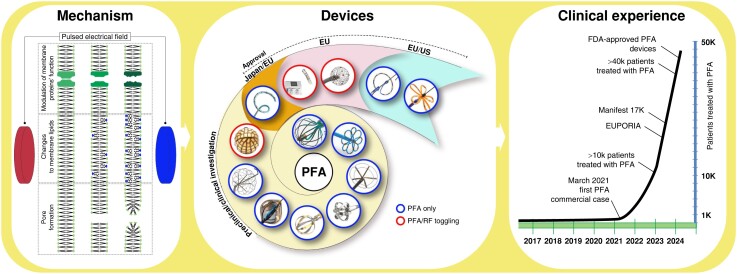

## Introduction

Catheter ablation is a well-established therapy option for rhythm control in patients with symptomatic atrial fibrillation (AF).^1^ Thermal-based energy sources such as radiofrequency current (RFC) or cryoballoon/laser balloon have shown to be reasonably safe and effective in this indication.^2–5^ However, treatment success remains limited, with single-procedure success rates of up to 80%. Furthermore, rare but significant energy-related complications to surrounding tissues, such as oesophageal injury or phrenic nerve palsy, may occur.^6–9^ Pulsed field ablation (PFA) is a novel, largely non-thermal ablation modality based on the process of irreversible electroporation (IRE). It holds the promise of creating transmural, durable ablation lesions while reducing the risk of collateral damage. Electroporation has a long-ranging history as a treatment modality in medical fields like oncology. In the 1980s, direct current shock was used for ablation of cardiac arrhythmias,^10^ but due to significant complications such as arching and barotrauma of these early technologies, as well as the success of the emerging RFC technology, PFA was no longer clinically pursued.

In the last decade, pre-clinical studies have shown that PFA in its modern form has the potential to create tissue-selective ablation lesions with short, high-energy electrical pulses that facilitate cell death while avoiding thermal energy source-related side effects.^11–13^ First in-human PFA trials demonstrated safe and efficient AF ablation procedures with high rates of both immediate pulmonary vein isolation (PVI) and durability on invasive re-mapping, resulting in comparably low rates of arrhythmia recurrence.^14–17^ As a result, various catheter platforms using electroporation have been developed and tested for catheter ablation of cardiac arrhythmias, mainly AF. We review the methodology of PFA and the pre-clinical and clinical data and discuss future directions.

## Mechanisms of electroporation

When a cell is exposed to an electric field, e.g. by delivering electric pulses to the tissue through a catheter, a transmembrane voltage is induced across the cell membrane. This is known as pacing in the field of cardiac electrophysiology. Even a relatively small signal will trigger depolarization or action potential. If high-voltage pulses are applied, the cell plasma membrane will undergo electroporation. Electroporation is a phenomenon that describes the transient increase of membrane permeability for ions and molecules otherwise deprived of transmembrane transport mechanisms. Induced transmembrane voltage in the order of several 100 mV is sufficient to cause pore formation in the lipid domain that leads to lipid oxidation but can also cause protein damage.^18–20^ Electroporation results in a transient increase of membrane permeability, which after some time recovers, and the cell can survive. Reversible electroporation as often referred to this phenomenon has been used in gene transfer and drug delivery for decades.^21,22^ Membrane electroporation and consequently membrane permeability (even if only transient) may cause the cell to die. This IRE has gained enormous attention,^23^ and it seems it offers an unprecedented speed and safety when it comes to cardiac ablation.^14,24^

**Figure 1:**
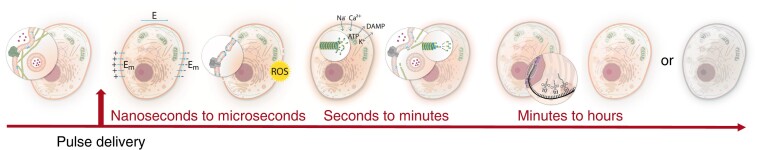
When a cell is exposed to sufficiently high electric field, by delivery of high-voltage electric pulses to tissue, a transmembrane voltage is induced, which leads to pore formation, lipid oxidation, and protein damage, rendering cell membrane permeable. Due to increased membrane permeability, ions and molecules flow in and out of the cells including Na^−^ and K^+^ ions that are depolarizing cell and Ca^2+^, which all results in increased intracellular Ca. At the same time, DAMP molecules—known to trigger immune response in tissue—leak out of the cell. Inflow of Ca can trigger other secondary processes like contraction and disassembly of cortical cytoskeleton and contributes to ATP depletion. Following membrane resealing, its conductivity still remains somewhat larger than before electroporation. The cell struggles to regain its homoeostasis by triggering its repair mechanisms, including heat shock proteins known also as chaperons required in protein (re)folding. Cell death depends on intensity of electroporation and may occur rapidly/immediately after exposure to electric pulses or can occur after several hours or even day(s). The processes described are highly dynamic and start in nanoseconds to microseconds and last for hours. ATP, adenosine triphosphate; DAMP, danger-associated molecular pattern; ROS, reactive oxygen species.

Even though cell death due to electroporation is mechanistically not fully understood, it was demonstrated that following plasma membrane electroporation, the cell may recover membrane integrity but die within the next few hours^25^ (*Figure 1*). Cell death due to electroporation is most often ascribed to adenosine triphosphate exhaustion, protein damage, e.g. ion channels, and calcium overload and is more generally ascribed to loss of cell homoeostasis.^26^ Cardiac ablation by electroporation is often described as non-thermal and cardiac tissue selective.^17^ The non-thermal nature of this energy and its selectivity must be taken with a grain of salt though. Namely, if high-voltage pulses are delivered to tissue through catheters in the blood pool (1500–3000 V), a considerable current will be drawn from the generator (10–20 A)—some heating is inevitable. It will be proportional to the product of the local electric field and current density—like RFC ablation: the non-thermal nature comes from the fact that the cells are killed by electroporation rather than by heat.^27^ It takes microseconds or even less to cause electroporation. In such a short time, the energy is too low to considerably heat up the tissue. However, if many repetitive pulses are delivered (with high duty cycle), the energy deposited in the tissue may cause a significant temperature increase locally and the ‘non-thermal’ property of PFA will vanish. Of note, cells that may be situated in high electric fields (e.g. erythrocytes) may become electroporated.^28,29^

Parameters such as pulse amplitude, pulse width, number of pulses, polarity (biphasic or monophasic), and pulse cycle—altogether ‘waveform,’ but also, catheter shape/geometry and distance of the tissue/cells from the catheter/electrodes since the electric field drops rapidly with the distance from the electrode/catheter—are parameters that affect and determine effective lesion creation.^23,30^

**Figure 2:**
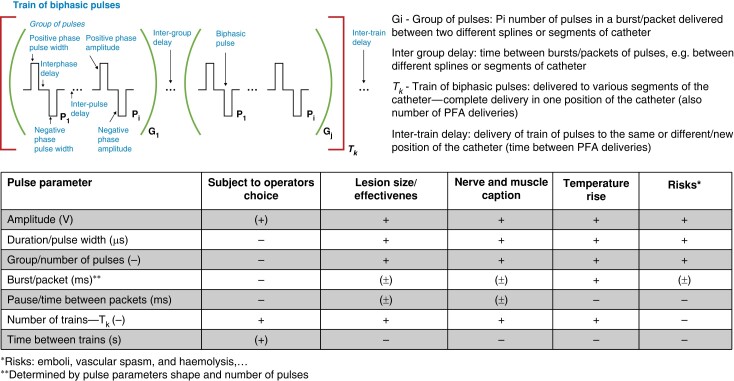
Most of pulse parameters were selected by manufacturers during the development, testing, and optimization of the system (pulse generator and catheter) and may differ from PFA system to PFA system. These pulse parameters (pulse amplitude, pulse width, number of pulses, interphase, and interpulse delay) may have profound effect on effectiveness and risks: lesion size, electrode/catheter breakdown risk, bubble formation, temperature rise and potential thermal damage, electrical arcing, barotrauma risk, and emboli risk and are therefore most often not available to the operator to change, i.e. are locked. In most system, there is little to no choices left to the operators and these in a limited range and with some limitations. There are but few parameters left that can be controlled during PFA procedure. Most often subject to variations and choices: *T_k_*—number of trains/PFA deliveries, time between deliveries (usually longer times are preferred to avoid temperature rise due to stacking effect), and pulse amplitude (voltage). It must be however emphasized that effects and risks depend in often non-linear relation to pulse parameters and that many of the outcomes (effects and risks) depend on multiple parameters. Optimization of the waveform and pulse parameters can be considered a multi-parameter optimization with conflicting requirements. PFA, pulsed field ablation.

Most of pulse parameters were selected by manufacturers during the development, testing, and optimization of the system (pulse generator and catheter) and may differ from PFA system to PFA system (*Figure 2*).^23,30^

The first devices were developed for relatively thin-walled atria and more specifically for PVI where lesion depth of few millimetres suffices,^31^ but targeting thicker ventricle may be a greater challenge.^32^ It seems that lesion depth, among other parameters, is related to waveform and amplitude largely depends on vectoring; i.e. bipolar PFA may be less efficient to create deep lesions compared with monopolar delivery.

As mentioned above, although PFA is considered non-thermal and selective, none of this is absolute and general. Electroporation is used in bacterial inactivation, and also, bacterial transformation by introducing genes of interest, similar cells, and tissues of all origins can be effectively electroporated—with no exception.^33^ Although *in vivo* tissue selectivity seems convincing, there is little evidence available supporting cell electroporation selectivity in vitro.^34,35^ Energy-specific mechanisms regarding lesion formation are summarized in *Table 1*. Side effects relevant in thermal energies may not be relevant for PFA (if kept non-thermal), but other unknown side effects may emerge. Although coronary artery spasm came as a surprise,^36,37^ the effects of electric pulses on blood, perfusion, and vasculature are known.^38–40^ Similar to cardiac pacing, electric pulses depolarize neurons—used for many years in electrical stimulation; it should be expected that the cardiac autonomic nervous system (in particular N. vagus) will be stimulated and may be responsible for bradycardia observed after PFA.^41^ Reversible stunning of phrenic nerves was studied *in vivo* and observed in clinic cases.^42,43^ Of note, known side effects can be worsened with more aggressive waveforms, and others may still need to be discovered.^44^

**Table 1:** Comparison of various attributes of different ablation energies and PFA cardiac selectivity

Ablation modality	Energy delivery and propagation	Cell damage	(Micro)vasculature	Extracellular matrix	Healing
Radiofrequency ablation (thermal)	Volumetric heating and thermal diffusion	Denaturation of proteins (indiscriminate)	Highly thrombogenic, indiscriminate thermal damage	Coagulation	Delayed, from the periphery
Cryoablation (freeze–thaw)	Thermal diffusion	Crystal formation–cell membrane damage; osmotic imbalance	Clogging and damage of microvasculature	Preserved	Delayed, from the periphery
Pulsed field ablation (electroporation)	Field effect and volumetric heating	Cell membrane damage	Transient reduction of blood perfusion	Preserved	Enabled/facilitated

PFA, pulsed field ablation.

**Figure 3:**
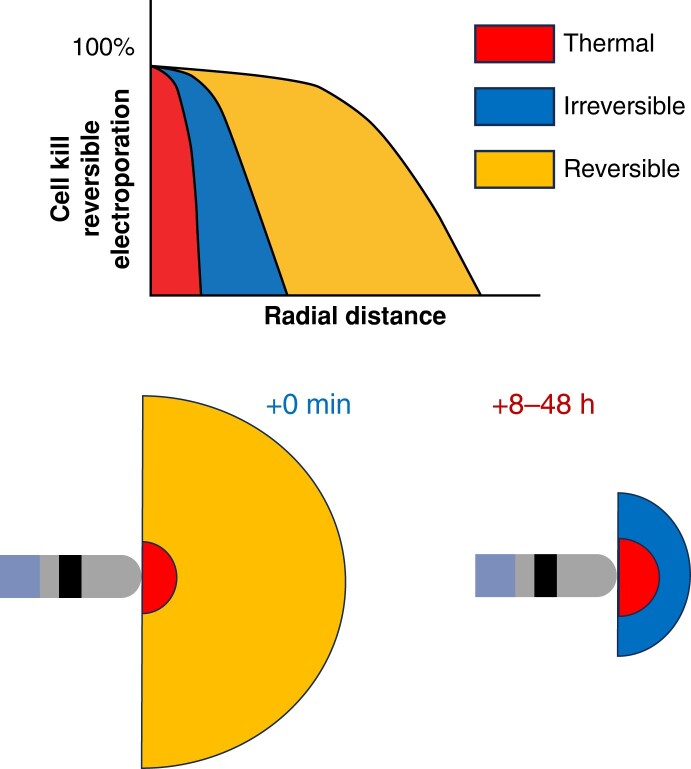
Closest to the catheter/electrode, the electric field and current density are the highest and decrease rapidly with the (radial) distance. This means that cells depending on the distance from the catheter will be exposed to higher or lower electric field and will be ‘electroporated’ to a different extent. Those exposed to highest electric field may die immediately, those exposed to somewhat lower intensity may die with a considerable delay, whereas cells exposed to even lower electric field may be electroporated only transiently and may recover. During the period of time of reversible electroporation, these cells may not be able to produce and conduct action potentials and will be electrically silent (noted as absence of electrograms). With high number of pulses of high voltage, inevitably tissue will be heated by Joule heating, which may cause some cell (closest to the catheter) to be thermally damaged.

Pulsed field ablation currently has a major drawback as intracardiac electrograms disappear virtually with the first pulse delivery. The traditional guiding of intracardiac electrocardiogram (ECG) signals in cardiac electrophysiology thus appears less important. It was shown before that electroporation increases membrane permeability for ions (and other molecules), which in other words ‘increases membrane conductivity’.^45^ Increased membrane conductivity alone in excitable cells can cause membrane depolarization and spontaneous contractions and trigger action potentials or can render cells unexcitable for a period of time.^20,46^ Upon the resealing of the membrane and regain of membrane selective permeability, the cells again behave ‘normally’.^46–48^ This is a clear consequence and result of reversible electroporation and has been shown theoretically by using a simple Hodgkin–Huxley-type model, which was shown both *in vitro* on a single cell and monolayer of cells mimicking cardiac tissue.^49^ For easier understanding, given a focal catheter, the lesion achieved by PFA will have a central irreversible zone surrounded by a rim of reversibly electroporated cells. The final resulting PFA lesion will be formed with delay (*Figure 3*). This also means that despite the impressive acute procedural efficacy, i.e. acute PVI reported in clinical studies, using PFA may not necessarily translate into superior long-term effects.^14^

### Role of contact force

Compared with thermal energy sources and based on available pre-clinical work, PFA lesion size/depth is much less dependent on contact force.^50–53^ This, together with the ability to create lesions even in the absence of direct contact with tissue (electrically conductive path is provided by relatively high conductivity of blood),^54^ provides the basis to create contiguous lesions even in trabeculated areas. In summary, PFA catheter-to-tissue contact is desired but appears to be less important compared with RFC energy.

## Technologies

**Figure 4:**
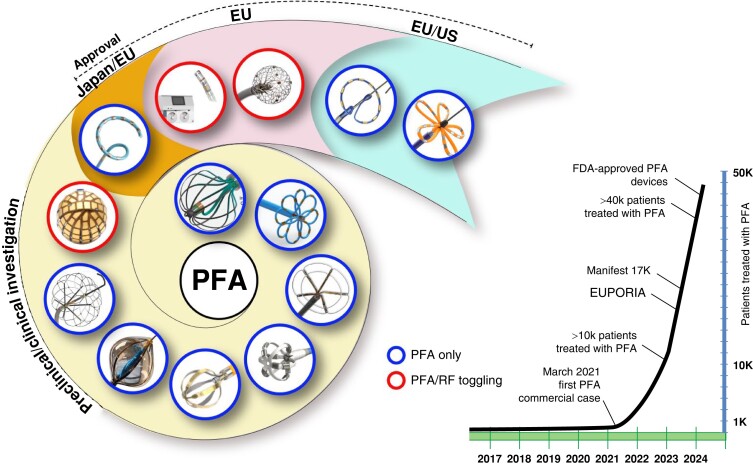
The evolution of novel and contemporary PFA devices. Recent approval resulted in rapid real-world adoption. For more details, see text and *Tables 2 and 3*. PFA, pulsed field ablation; RF, radiofrequency.

There are numerous PFA catheters, designs, and generators under development and investigated in pre-clinical and clinical studies (*Tables 2* and *3* and *Figure 4*). Due to the clinical need, most catheters so far have been designed to treat atrial arrhythmias but ventricular ablation will be in focus in the near future. In brief, contemporary catheters can be divided into (i) circumferential PVI tools or (ii) point-by-point ablation devices. Within this latter group, novel-specific (large/intermediate footprint) devices or conventional RFC catheters deploying PFA as an alternative energy source may be used. Due to the rapid evolution in the field, not all PFA devices can be discussed but different categories of catheters and stages of evolution are shown (*Figure 4*).

**Table 2:** Overview of contemporary circumferential PVI tools

	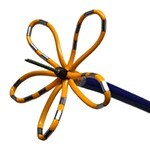	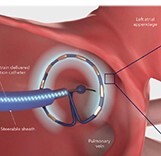	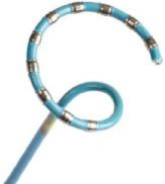	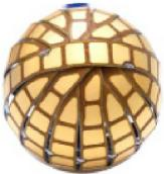	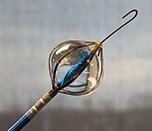	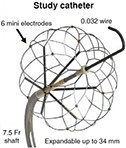	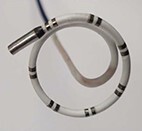	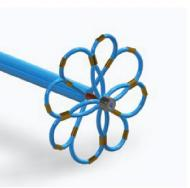	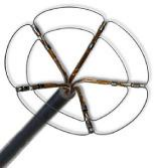
	Farapulse™ Boston Scientific	PulseSelect™ Medtronic	Inspire™ Biosense Webster	Globe PF™ Kardium	Volt™ Abbott	Sphere 360™ Medtronic	Adagio Adagio	Lotos™ Lifetech	CellFX nsPFA™ Pulse Bioscience
Diameter	31/35 mm	25 mm	25–35 mm	30 mm	28 mm	34 mm	25 mm variable	28/31 mm	30 mm
Size	12F	9F	8.5F	16F	13F	8.5F	8.5F	12F	11F
Over the wire	Yes	Yes	No	No	Yes	Yes	No	No	No
PVI	++++	++++	++++	++++	++++	++++	+++	++++	++++
Non-PV lesions/versatility	+++	++	++	++	+	+	++	++	++
Clinical experience	+++++	+++	++	+	+	++	+	No	+
Ablation mode	Bipolar	Bipolar	Bipolar	Bipolar	Bipolar	Monopolar	Bipolar	Bipolar	Bipolar
Dedicated 3D mapping	No	No	Yes	Yes	Yes	Yes	No	No	No
Approval	EU/USA	EU/USA	EU/Japan	No	No	No	No	No	No

Several systems are currently under investigation; thus, scientific references are not always available. The assessment of clinical applicability is based on the authors’ opinion. For more details, please see text.

PF, pulsed field; PFA, pulsed field ablation; PVI, pulmonary vein isolation.

**Table 3:** Overview of focal point-by-point large/intermediate footprint PFA catheters and a specific generator enabling PFA using conventional RF catheters

	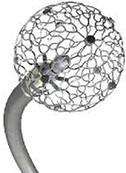	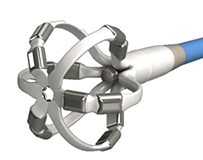	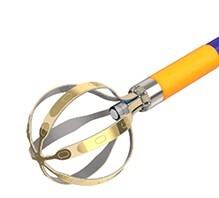	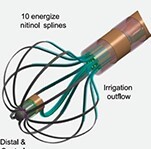	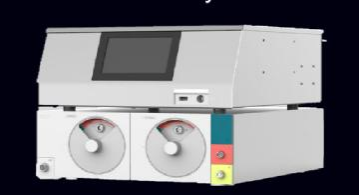
	Sphere 9™ Medtronic	Omnypulse ™ Biosense Webster	Faraflex ™ Boston Scientific	LFC Centauri	Centauri
Diameter	9 mm	12 mm	10 mm	10 mm	3.5–4 mm
Size	8F	7.5F	8F	8.5Fs	8F
Catheter deflection	Bidirectional	Bidirectional	Bidirectional	Bidirectional	Unidirectional/bidirectional
PVI	++++	+++	+++	NA	+++
Non-PV lesions/versatility	++++	++++	++++	NA	++++
Clinical experience	++++	+	+	No	++
Ablation mode	Monopolar	Bipolar	Bipolar Monopolar	Monopolar	Monopolar
PFA/RFC	Yes/Yes	Yes/No	Yes/No	Yes/No	Yes/Yes
Dedicated 3D mapping	Yes	Yes	Yes	multiple	multiple
Approval	EU	No	No	No	EU

Several systems are currently under investigation; thus, scientific references are not always available. The assessment of clinical applicability is based on the authors’ opinion. For more details, please see text.

PFA, pulsed field ablation; PVI, pulmonary vein isolation; RF, radiofrequency; RFC, radiofrequency current.

## Categories

Circumferential PVI devices (*Table 2*)

Focal point-by-point PFA devices (*Table 3*)

- Large/intermediate footprint catheter, 3D electro-anatomical (EA) mapping system integration
- Conventional RFC catheter, specific generator offering RFC and/or PFA

## Circumferential pulmonary vein isolation devices

### Farapulse™: pentaspline device

**Figure 5:**
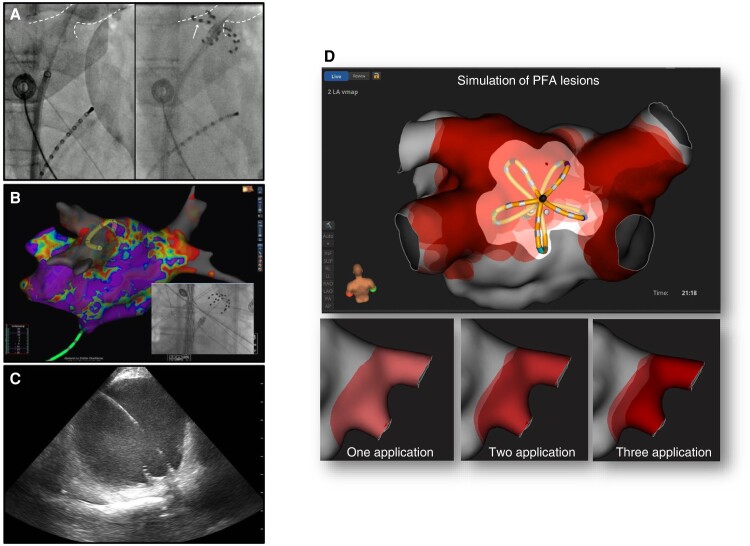
Pulsed field ablation practical considerations. Pulsed field as an energy form is less contact force sensitive, since the electrical field spreads into tissues surrounding the catheter. However, pre-clinical data show that tissue contact has a direct effect on lesion depth and durability. Currently, catheter visualization, navigation, and contact assessment are primarily based on fluoroscopy, intracardiac echocardiography, or simplified impendence-based 3D mapping. In future, sophisticated concepts regarding catheter navigation and contact assessment will evolve (*Figure 4*). If deep sedation instead of general anaesthesia was used, PFA typically stimulates skeletal muscles and the phrenic nerve. In this scenario, a stabilizing guidewire may be beneficial reducing the risk of device dislocation but needs to balanced vs. the risk of mechanical trauma. (*A*) The pentaspline device position can be assessed in comparison with selective PV angiograms. In addition, the interaction between splines and tissue adds valuable information regarding local contact (arrow). (*B*) Alternatively, the pentaspline devices can be visualized within a 3D mapping system with impedance-based catheter localization. However, the device is displayed only as a circular structure, reducing catheter positioning accuracy. (*C*) Real-time catheter localization may be also assessed using intracardiac echo: besides embedded costs, this seems to be a viable alternative to reduce X-ray exposure (courtesy of Bart Moulder). (*D*) Future 3D mapping integration and lesion visualization of repetitive pentaspline applications (courtesy of Melanie Gunawardene). PFA, pulsed field ablation.

This ablation device consists of five splines carrying 20 electrodes and is available in two sizes (maximal diameter: 31 and 35 mm), which can be seamlessly changed from the so-called flower to a basket configuration (Farapulse™, Boston Scientific).^55^ Pulsed field ablation delivery consists of trains of bipolar, biphasic stimuli using electrical field strengths ranging from 1.8–2.0 kV with fixed 2.5 s duration. The device is able to pace and record cardiac electrograms for simple mapping and electrophysiological manoeuvres. The catheter is navigated through a steerable sheath (Faraflex, ID 13.6F), positioned at the PV antrum and stabilized via a guide wire anchored in a PV branch. After a pair of applications in flower and basket or olive configuration, the catheter is rotated to ensure a circumferential transmural lesion. Navigation and ablation at non-PV sites such as the left atrial (LA) roof or posterior wall are technically feasible.^56^ Device navigation is typically guided by fluoroscopy, intracardiac echo, or 3D mapping (*Figure 5*).^57^ Currently, contemporary 3D mapping systems offer only very basic catheter visualization. Sophisticated future 3D map features are under development, which may allow for precise device visualization, spline selection, catheter-to-tissue contact assessment, and PFA lesion simulation (*Figure 5D*). The Farapulse™ device is Ce marked and has recently received FDA approval.

### Varipulse: variable loop circular catheter

This PFA system represents an evolution of the former circular irrigated RFC ablation platform.^9^ The VARIPULSE™ catheter (8.5F bidirectional, Biosense Webster) is sizeable from 25–35 mm diameter, fully integrated into a 3D mapping system and incorporates proprietary waveform technology to deliver short-duration, high-voltage bipolar biphasic pulses.^9^ The catheter is combined with the Trupulse generator. Each pulse is delivered as a square wave with positive and negative phases. Briefly, PFA is applied in a bipolar configuration with an energy of 1.8 kV. The trains are microsecond-long biphasic pulses resulting in a total application duration of ≈250 ms. The catheter is rotated to ensure overlapping lesions. The generator can also be programmed to deliver energy to specific electrode pairs and to adjust energy delivery based on the clinical needs. The device has obtained Ce mark.

### Pulse select: fixed-sized multi-polar circular device

This PVI PFA system represents an evolution of the former circular RFC ablation platform (Medtronic Pulse Select™).^24^ The 9F catheter is navigated over the wire through a 10F steerable sheath. It consists of a circular array (25 mm diameter, 20° tilt) including nine gold electrodes. The novel PFA generator delivers biphasic, bipolar waveforms (1.5 kV, on the order of milliseconds per delivery). The catheter is rotated to ensure overlapping lesions. All deliveries are automatically gated to the ventricular refractory period.

In principle, impedance-based visualization of this device within a 3D EA mapping system is feasible, but there is no sophisticated image integration available at this point in time. This device is Ce marked and is the first that has recently received FDA approval.

### Globe PF™: spherical mapping and ablation device (Kardium)

The Globe™ Pulsed Field Mapping and Ablation System (Kardium Inc.) is a 30 mm array consisting of 16 ribs with a total of 122 gold-plated electrodes that can map intracardiac electrograms, pace, and measure tissue contact and temperature. The latest generation of this spherical catheter also allows for PFA delivery.^58^ Up to 64 electrodes can be selected to customize the PFA ablation path (proprietary waveforms, biphasic, bipolar, single train, biphasic, and bipolar delivery). The catheter (16F) is inserted into the LA through a specifically designed bidirectionally deflectable sheath. In the LA, the coiled ribs fan out to form a spherical array. The pulsed field (PF) generator waveform uses propriety pulse parameters and is not modifiable by the operator (1.7 kV, microsecond PF pulses per application). The system allows to create a 3D electroanatomic reconstruction including voltage, activation, and contact mapping.^59^ This device is currently under clinical investigation.

### Combined pulsed field ablation and ultralow cooling

This platform has been originally designed for ultralow cooling and enables both single-shot PVI and focal ablation (Adagio iCLAS™). The catheter (8.5F, 20 poles, central lumen, and stylet) requires a steerable sheath. The combination of PFA and cryothermal ablation may lead to a favourable thermal profile, reduced muscle contractions, and microbubbles while extending lesion.^60^ A prospective clinical study (parallel trial) is currently randomizing persistent AF patients in Canada and Europe [PFA vs. PFA and cryothermal ablation: PVI + posterior wall isolation and cavo-tricuspid isthmus (CTI) ablation according to the operator]. No clinical data have been published at this point in time.

## Focal pulsed field ablation devices

This family of ablation devices combines focal point-by-point large/intermediate footprint PFA integrated into novel or contemporary 3D EA mapping systems to deploy individualized lesions based on strategies beyond empiric ablation. Most of these PFA-only catheters are under development or clinical evaluation lacking large-scale human experience (*Table 3*).

### AFFERA™–Sphere 9™ toggling pulsed field ablation and radiofrequency current ablation integrated into a 3D mapping system

This novel ablation and mapping system uses a 9 mm lattice tip catheter (Sphere 9™) covered with nine mini electrodes, which is fully integrated into a high-resolution 3D mapping system (AFFERA™, Medtronic). All electrodes record local temperature and electrograms allowing for activation mapping. The catheter was originally designed to deploy high-power temperature-controlled radiofrequency ablation resulting in lower current density due to the large footprint lattice design. The generator allows for toggling between monopolar PFA from the catheter tip to indifferent electrodes and irrigated radiofrequency ablation.^15^ Lesion depth can be increased with repetitive PFA applications.^61^ Therefore, this device allows for point-by-point PVI as well as individualized ablation. Both energy modalities can be adjusted according to the physician's preference. Initial human experience indicates safe and durable ablation on the atrial level. More data regarding workflow considerations such as general anaesthesia or deep sedation are needed. This device is Ce marked but not FDA approved.

### Pulsed field ablation generator connected to conventional radiofrequency current catheters

CENTAURI represents the first platform technology (Galvanize Therapeutics, San Carlos, CA, USA) enabling PFA delivery via commercially available established focal RFC ablation catheters. This type of PFA combines contemporary contact force RFC catheters and 3D EA mapping systems with a novel PFA generator. The generator delivers biphasic, monopolar PF energy at three selectable energy settings (19, 22, and 25 A) through the tip electrode of the ablation catheter. The system permits connectivity of compatible focal ablation catheters and their mapping systems that synchronizes PFA delivery to the R wave.^62^ Initial clinical data indicate feasibility and safety.^62,63^

The device is Ce marked for the treatment of atrial arrhythmias.

In addition, clinical studies are currently investigating the safety and efficacy of conventional contact force RFC catheters using novel dual energy generators toggling between PFA and RFC ablation.

## Clinical data

### Available pulsed field ablation technology

Numerous different ablation devices for PFA have been designed and are currently under clinical investigation. After recent approval, rapid adoption has been observed in the real world (*Figure 4*).

#### Performance

The above-mentioned technologies were primarily designed to achieve durable PVI. Hence, the rate of acute and durable PVI may be considered a marker of performance.

In pre-marketing studies, the Farapulse™ pentaspline catheter was evaluated for performance in *protocol-mandated* re-mapping studies. Acute PVI was achieved in 475/475 PVs (100%), and PVI durability was noted to be 96 and 84.8% on per PV and a per patient level, respectively.^17^

More re-mapping data stem from clinically indicated repeat procedures in patients with symptomatic atrial tachycardia (AT)/AF recurrences. In a single-centre experience, durable PVI was reported to be 90.9% showing a significant difference in favour of the 31 mm pentaspline catheter as opposed to the 35 mm device.^64^ Multi-centre experience from the EU-PORIA registry and the ADVENT study found PVI durability in 72 and 64% of all PVs, respectively.^14,57^ It appears that the anterior aspect of the lateral PVs is prone to recovered LA to PV conduction in particular when the 35 mm pentaspline catheter was used. Future studies may further investigate alternative dosing regimens as well as ways to improve catheter-to-tissue contact.

Accordingly, re-mapping data after PVI with the variable loop PFA catheter showed durable PVI rates between 10 and 30.8% depending on the PFA waveform.^65^ In the PULSED-AF study, 41/300 patients underwent a repeat ablation but PVI durability data have not been disclosed.^66^

Protocol-mandated re-mapping was also performed in a study using a PFA system employing commercially available contact force tip catheters. After having optimized the workflow, per PV durable isolation rates ranged from 84 to 92%.^62^

Similarly, a novel lattice tip catheter that allows for toggling between PFA and thermal ablation was evaluated for its performance. During protocol-mandated re-mapping, a 97% durable PVI rate was noticed using the optimized waveform.^15^

Both the focal and the lattice tip catheter enable the operator to apply linear ablation concepts. With the latter, acute bidirectional conduction block was achieved in all patients at the LA roof, the mitral isthmus, and the CTI mostly using both energy modalities. For the linear atrial lesions, the overall durability for the LA roof, mitral isthmus, and CTI lines was 82, 68, and 87%, respectively.

Most recently, it was reported that repeat PVI and LA anterior line ablation can be carried out with a standard tip ablation catheter using PFA. Bidirectional conduction block was achievable in 84% of attempts.^67^

#### Safety

In first in-human studies, excellent procedural safety was reported. Serious adverse events occurred in 0–2.5% of patients. In particular, no energy-specific complications such as thermal oesophageal injury or phrenic nerve palsy were reported.

A similar favourable safety profile (2.1% procedural complications) was observed in the ADVENT trial. However, it should be noted that one patient died from a pericardial tamponade.

After commercialization, the MANIFEST PF survey was the first study to report complication rates for the pentaspline PFA catheter on a centre level.^37^ Implementing workflow changes and after having passed initial learning operator curves, the rate of procedure-related complications decreased (MANIFEST 17k) substantially. Pericardial tamponade and stroke were reported to be as low as 0.36 and 0.12%, respectively.

In an attempt to determine the thromboembolic risk profile of various catheters, cerebral magnetic resonance imaging (MRI) was performed in asymptomatic patients. The rates of silent cerebral lesions (SCLs) varied from 3 to 19% for the pentaspline catheter.^14,16,17,68^ In the Pulsed AF trial, 45 patients underwent pre-procedural and post-procedural MRI, and SCLs were detected 9% of patients after the ablation. In the Inspire study, the SCL rate was 67 and 12% before and after workflow optimization.

Similarly, very rarely energy-specific complications such as coronary spasm or ablation-induced haemolysis were described. The latter seems to be correlated to the number of PFA applications, and 70 applications per procedure have been identified as a critical threshold for clinically relevant haemolysis. As a consequence, acute kidney injury was incidentally noted, which can be mitigated by infusion of large amounts of normal saline.^69,70^ Still, in the light of rapid adoption of novel PFA devices, more data are required to understand the role of this complication in the real world. For sure, more PFA dosing data are needed and ablation should be carefully guided by the clinical need.

It seems that the occurrence of coronary spasm is closely related to the distance between the ablation catheter and vessel. In a systematic study, coronary angiograms were performed during PFA applications at various locations.^71^ While no coronary spasm was observed during ablation at the PVs, ablation at the CTI led to coronary spasm in all cases. Coronary spasm could be resolved by intracoronary application of nitroglycerin and could also be prevented by intravenous drug application.

Similarly, ablation at the mitral isthmus may be associated with spasm of the circumflex artery in a considerable number of cases and an inferior ablation line may help to avoid this complication.^72^ In another observational study, mitral isthmus ablation with the pentaspline PFA catheter led to a high acute success rate and was associated with 4.4% non-fatal coronary spasms.^73^ To date, no relevant PV stenosis has been reported. Recently, a sub-analysis of the ADVENT trial was published without any evidence for PV stenosis in PFA-treated patients.^74^

Most importantly, the risk for thermal oesophageal injury seems to be eliminated. In several studies, oesophageal temperature monitoring and post-procedural endoscopy revealed no evidence for significant heating or mucosal injury.^16,75^ Moreover, selective sparing of oesophageal tissue by PFA ablation at the posterior LA wall was elegantly demonstrated using MRI.^76^

#### Efficacy

Several studies reported freedom from atrial arrhythmia after PFA ablation. When comparing outcomes between different PFA ablation devices, it has to be noted that follow-up strategies (i.e. intensity) may vary substantially. In the ADVENT and Pulsed AF pivotal trials, weekly trans-telephonic ECG submissions and 72 h Holter at varying time points were performed. Freedom from arrhythmia was estimated to be 73.3 and 66.2% in ADVANTAGE and Pulsed AF, after 12 months, respectively.

In real-life clinical registries with less intense rhythm, monitoring rates of freedom from arrhythmia were 80–82 and 66–72% for patients with paroxysmal and persistent AF, respectively, which are in line with thermal ablation.^77–81^ Of note, more real-life long-term outcome data are needed.

### Impact on the autonomic nervous system

Due to its relative tissue selectivity, PFA may not permanently affect the cardiac autonomic nervous system. In fact, in an elegant study, extracardiac vagal stimulation was used to compare the effects of PFA and irrigated RFC-guided PVI on the sinus node and atrioventricular node, respectively.^82^ It showed that RFC ablation led to a significant attenuation of the response to extracardiac vagal stimulation. Oppositely, this response was much less common after PFA ablation and effects rapidly returned to baseline. The authors concluded that cardiac vagal response is preserved in patients treated with PFA. This observation is supported by further studies analysing serum markers of neuronal damage after PFA and cryothermal ablation.^41,83^ Both studies found a significantly lower release of S100B after PFA ablation.

However, efforts are made to design PFA devices that specifically electroporate cardiac ganglion cells to modify the cardiac autonomic nervous system with minimal harm to cardiac myocytes.^84^ First in-human data were recently published providing promising data on the feasibility and safety of ganglionic plexi ablation during cardiac surgery.^85^

## Future clinical implications

The integration of PFA catheters with available 3D mapping systems is expected to become reality in the near future. It may allow more precise PFA and thereby reducing the risk of gaps. So far, only AFFERA™ and very recently Varipulse™ have been offering 3D mapping capabilities. The Farapulse™ catheter integration into a specific 3D mapping system is under development and expected to be available soon (*Figure 5D*). Specific software may provide information on contact, spline labelling, and potentially predicting lesion extent, transmurality, and durability.

AFFERA™ and Sphere9™ use a very modest but detectable increase in electrode temperature during deliveries as a surrogate for tissue contact. Presently, numerous treatment planning software have been developed for IRE and electrochemotherapy (i.e. combination of electroporation and chemotherapy) in oncology.^86–88^ It is currently unclear whether meticulous refinement using advanced mathematical methodologies and computing^89^ is needed in catheter ablation of arrhythmias. One could argue that the safety profile of PFA allows for maximizing efficacy without compromising safety. Tailored strategies could be advantageous, particularly if accounting for tissue information such as thickness or fatty/fibrotic infiltration. Fat, by acting as an insulator for electricity, is a major limiting factor for RFC ablation, whether this may be less relevant for PFA needs to be assessed.

### Developments in atrial fibrillation ablation

It should be first acknowledged that PVI is likely to be the only needed strategy for the vast majority of paroxysmal AF patients. Initial clinical PFA experience suggests high rates of durable PVI and less operator dependency. This may contribute to future increased and predictable success rates.^15,17,57^ Ablation strategies in persistent AF are much less mature, with no consensus beyond PVI. It remains unclear whether one strategy could fit to all clinical scenarios. Pulsed field ablation may help to understand whether AF recurrences were due to insufficient mapping or ablation. Importantly, as PFA is easy to deploy and powerful, the best ablation strategy is the one restoring sinus rhythm at the least tissue cost.

The Farawave™ catheter has been designed for PVI and is not optimized for linear lesion delivery. Interestingly, it performs technically well in posterior LA wall ablation. Reconnections through that posterior wall area are rare when compared with RFC.^90^

The remarkable absence of oesophageal lesions with this system allows to deliver the lesion in a very low position, where the septo-pulmonary bundle is no longer an issue. It should, however, be noted that focal catheters such as Sphere 9™ may be more suitable for tailored linear lesions.^15^ This is probably even more true for the mitral isthmus line. The durability of the Farapulse™ linear block is suboptimal, even if combined with Marshall vein alcohol injection. In addition, post-mortem studies have unveiled diverse configurations of myocardial tissue surrounding the CS and/or Marshall bundle, forming endocardial–epicardial bridges—surrounded by fat—across the mitral isthmus line and connecting with the LA.^91–93^

Providing safer, faster, and possibly more durable lesions, PFA may offer an opportunity in the future to explore major questions in AF ablation:

1. Pulmonary vein isolation responder vs. PVI non-responder in AF ablation
  - What is the real rate of PVI responder in paroxysmal and persistent AF?
2. Pulmonary vein isolation non-responder
  - What is the best ablation strategy?
  - Linear lesions: more safety and efficacy data, particularly for the mitral isthmus is needed (e.g. focal PFA within the CS)^15^
3. What is the role of tailored ablation in the real world?
4. What is the role of deep sedation vs. general anaesthesia?^94–96^

### Pulsed field ablation in trabeculated areas and right atrium

In the context of atrial ablation some atypical re-entries or focal targets include trabeculated areas. They are often difficult to ablate using RFC, most likely because of their complex 3D topography. Pulsed field ablation has the advantage of treating similar pouches and crests, as it affects tissue included in the treatment volume. It, therefore, offers a theoretical advantage, but this has to be confirmed in clinical practice. If ablation close to the phrenic nerve was required, PFA may become the preferred energy source.^97^ Using PFA for CTI is effective albeit with documented instances of adverse events like coronary artery spasm.^71,98^ Notably, proactive administration of intracoronary/intravenous nitroglycerin has been shown to significantly attenuate vasospasm, emphasizing a potential prophylactic measure.^71^ In summary, PFA within the right atrium offers advantages but necessitates a cautious appraisal of potential risks, encompassing coronary artery spasms and disturbances of the sinus node and/or conduction system.^99^

### Pulsed field ablation for VT ablation

**Figure 6:**
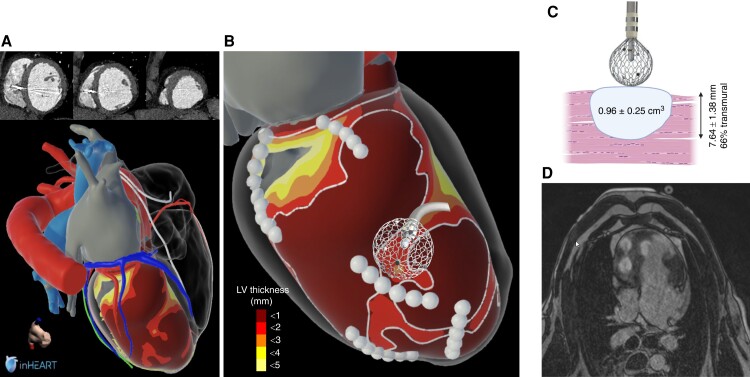
(*A*, *B*) VT ablation in the near future will take advantage of image integration combined with centimetric ablation electrodes positioned on CT channels (in between areas of block as circled in white) after registration in the navigation system. (*C* ) Illustrates pulsed field ablation lesions as imaged acutely after three deliveries placed in the LV septum of a sheep using the AFFERA system. Two additional lesions are visible on that acute late gadolinium enhancement MRI in the RV. Lesion size (*D*) is extracted from acute MRI. Such a lesion would allow a ‘single shot approach’ to block a channel. LV, left ventricle; RV, right ventricle.

Although PFA has proceeded to randomized trials in patients with AF, there are still sparse data on ventricular ablation with PFA, especially in infarcted myocardium.^100^ Dosing remains unclear and has to be studied for different devices and anatomic locations. At first sight, the benefits may be less for ventricular lesions where collateral damages are not so frequent. Radiofrequency current, particularly with AFFERA™, is producing lesions as deep as 12 mm, which would be critical for septal or mid/epicardial substrate, particularly when wall thickness is preserved or increased. Obviously, it will require more in-depth studies. Another aspect, probably not so obvious but really important, might be the ability of PFA vs. RFC to effectively ablate despite of scar tissue with fibrosis and fat infiltration. Radiofrequency current within heterogeneous scar tissue results in non-uniform tissue injury and unpredictable lesions regarding morphology and dimensions,^101^ possibly due to lower tissue temperatures. Demonstrating PFA efficacy in scar tissue will be key, as well as defining and refining dosing possibly based on image integration (*Figure 6*). Data on target substrate composition including tissue thickness and the presence of fibrosis and fat could impact dosing/number of PF deliveries. Repeated PFA application is the only way to increase both lesion depth and width with current systems.^61^ One interesting catheter innovation using PFA platforms represents the concept of focal large footprint/intermediate-sized ablation electrodes. Irrespective of the energy used, those ablation electrodes carry the potential to transect most post-myocardial infarction VT isthmuses with only one location, thereby reducing the risk for non-contiguous lesions and reconnections (*Figure 6*). Using those platforms could lead to more predictable and simplified post-myocardial infarction VT ablation.

### The potential clinical value of reversible electroporation

When low-intensity PF was applied *in vivo* to porcine atrial tissue, local electrogram amplitude immediately vanished but gradually recovered over several minutes, underscoring the reversible nature of these very short pulses. The application of reversible pulses resulted in transient PR prolongation and dose-dependent atrioventricular block, indicating their capacity to transiently impair electrical conduction.^47^ Notably, if low-intensity PFA was deployed in critical sites, reproducible termination of atrioventricular nodal re-entrant tachycardia and atypical atrial flutter was demonstrated. This should also be kept in mind when IRE is desired.^47^

### Limitation

Unresolved issues such as optimal energy settings, catheter stability, and the learning curve associated with this novel technology warrant further investigation. Long-term follow-up data and large-scale randomized controlled trials will be crucial for establishing the role of PFA in the real world.

## Conclusions

Pulsed field ablation represents a potentially disruptive technology in the field of cardiac electrophysiology, offering a potential paradigm shift in the treatment of arrhythmias. The accumulating body of evidence supports its efficacy and safety, positioning PFA as a more than just a viable alternative to traditional thermal ablation. However, more research and clinical experience is needed.

## Data Availability

The data underlying this article are available in the article.
