# Correction to: State-of-the-art pulsed field ablation for cardiac arrhythmias: ongoing evolution and future perspective

**DOI:** 10.1093/europace/euae182

**Published:** 2024-07-16

This is a correction to: Kyoung-Ryul Julian Chun, Damijan Miklavčič, Konstantinos Vlachos, Stefano Bordignon, Daniel Scherr, Pierre Jais, Boris Schmidt, State-of-the-art pulsed field ablation for cardiac arrhythmias: ongoing evolution and future perspective, *EP Europace*, Volume 26, Issue 6, June 2024, euae134, https://doi.org/10.1093/europace/euae134

In the originally published version of the manuscript, there were errors in Table 2. In second column “PulseSelect™ Medtronic”, line “Over the wire” should read: “Yes” instead of: “No”. And in the sixth column “Sphere 360™ Medtronic”, line “Clinical Experience” should read: “++” instead of “+”; and line “Dedicated 3D” should read: “Yes” instead of: “No”.

There were also errors in Table 3. In 3rd column “Faraflect™ Boston Scientific”, line “size” should read: “8F” instead of: ”12F”. In first column “Sphere 9™Medtronic”, line “Clinical experience” should read: “++++” instead of: “+++”.

**Table 2:** Overview of contemporary circumferential PVI tools

	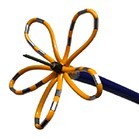	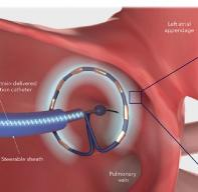	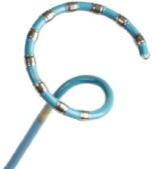	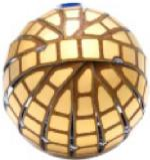	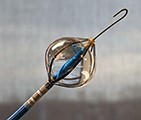	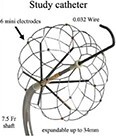	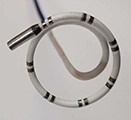	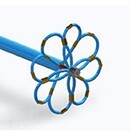	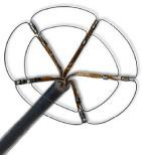
	Farapulse™ Boston Scientific	PulseSelect™ Medtronic	Inspire™ Biosense Webster	Globe PF™ Kardium	Volt™ Abbott	Sphere 360™ Medtronic	Adagio Adagio	Lotos™ Lifetech	CellFX nsPFA™ Pulse Bioscience
Diameter	31/35 mm	25 mm	25–35 mm	30 mm	28 mm	34 mm	25 mm variable	28/31 mm	30 mm
Size	12F	9F	8.5F	16F	13F	8.5F	8.5F	12F	11F
Over the wire	Yes	Yes	No	No	Yes	Yes	No	No	No
PVI	++++	++++	++++	++++	++++	++++	+++	++++	++++
Non-PV lesions/versatility	+++	++	++	++	+	+	++	++	++
Clinical experience	+++++	+++	++	+	+	++	+	No	+
Ablation mode	Bipolar	Bipolar	Bipolar	Bipolar	Bipolar	Monopolar	Bipolar	Bipolar	Bipolar
Dedicated 3D mapping	No	No	Yes	Yes	Yes	Yes	No	No	No
Approval	EU/USA	EU/USA	EU/Japan	No	No	No	No	No	No

Several systems are currently under investigation; thus, scientific references are not always available. The assessment of clinical applicability is based on the authors’ opinion. For more details, please see text.

PF, pulsed field; PFA, pulsed field ablation; PVI, pulmonary vein isolation.

instead of:

**Table 2:** Overview of contemporary circumferential PVI tools

	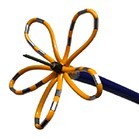	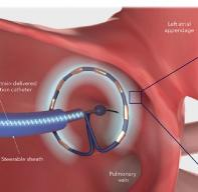	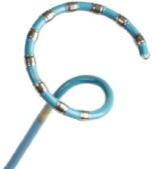	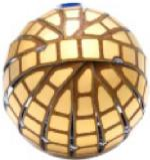	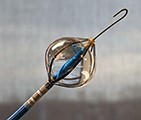	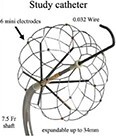	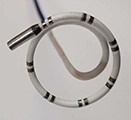	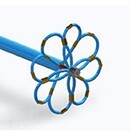	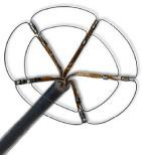
	Farapulse™ Boston Scientific	PulseSelect™ Medtronic	Inspire™ Biosense Webster	Globe PF™ Kardium	Volt™ Abbott	Sphere 360™ Medtronic	Adagio Adagio	Lotos™ Lifetech	CellFX nsPFA™ Pulse Bioscience
Diameter	31/35 mm	25 mm	25–35 mm	30 mm	28 mm	34 mm	25 mm variable	28/31 mm	30 mm
Size	12F	9F	8.5F	16F	13F	8.5F	8.5F	12F	11F
Over the wire	Yes	No	No	No	Yes	Yes	No	No	No
PVI	++++	++++	++++	++++	++++	++++	+++	++++	++++
Non-PV lesions/versatility	+++	++	++	++	+	+	++	++	++
Clinical experience	+++++	+++	++	+	+	+	+	No	+
Ablation mode	Bipolar	Bipolar	Bipolar	Bipolar	Bipolar	Monopolar	Bipolar	Bipolar	Bipolar
Dedicated 3D mapping	No	No	Yes	Yes	Yes	No	No	No	No
Approval	EU/USA	EU/USA	EU/Japan	No	No	No	No	No	No

Several systems are currently under investigation; thus, scientific references are not always available. The assessment of clinical applicability is based on the authors’ opinion. For more details, please see text.

PF, pulsed field; PFA, pulsed field ablation; PVI, pulmonary vein isolation.

Table 3 should read:

**Table 3:** Overview of focal point-by-point large/intermediate footprint PFA catheters and a specific generator enabling PFA using conventional RF catheters

	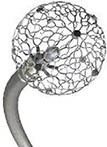	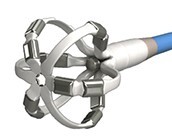	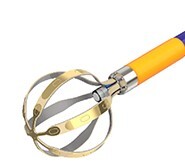	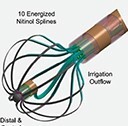	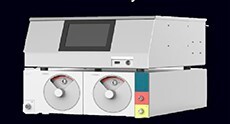
	Sphere 9™ Medtronic	Omnypulse ™ Biosense Webster	Faraflex ™ Boston Scientific	LFC Centauri	Centauri
Diameter	9 mm	12 mm	10 mm	10 mm	3.5–4 mm
Size	8F	7.5F	8F	8.5Fs	8F
Catheter deflection	Bidirectional	Bidirectional	Bidirectional	Bidirectional	Unidirectional/bidirectional
PVI	++++	+++	+++	NA	+++
Non-PV lesions/versatility	++++	++++	++++	NA	++++
Clinical experience	++++	+	+	No	++
Ablation mode	Monopolar	Bipolar	Bipolar Monopolar	Monopolar	Monopolar
PFA/RFC	Yes/Yes	Yes/No	Yes/No	Yes/No	Yes/Yes
Dedicated 3D mapping	Yes	Yes	Yes	multiple	multiple
Approval	EU	No	No	No	EU

Several systems are currently under investigation; thus, scientific references are not always available. The assessment of clinical applicability is based on the authors’ opinion. For more details, please see text.

PFA, pulsed field ablation; PVI, pulmonary vein isolation; RF, radiofrequency; RFC, radiofrequency current.

instead of:

**Table 3:** Overview of focal point-by-point large/intermediate footprint PFA catheters and a specific generator enabling PFA using conventional RF catheters

	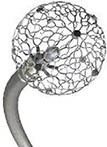	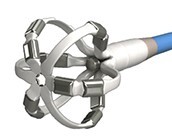	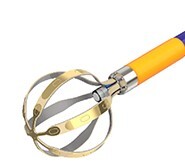	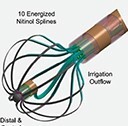	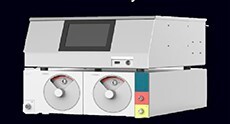
	Sphere 9™ Medtronic	Omnypulse ™ Biosense Webster	Faraflex ™ Boston Scientific	LFC Centauri	Centauri
Diameter	9 mm	12 mm	10 mm	10 mm	3.5–4 mm
Size	8F	7.5F	12F	8.5Fs	8F
Catheter deflection	Bidirectional	Bidirectional	Bidirectional	Bidirectional	Unidirectional/bidirectional
PVI	++++	+++	+++	NA	+++
Non-PV lesions/versatility	++++	++++	++++	NA	++++
Clinical experience	+++	+	+	No	++
Ablation mode	Monopolar	Bipolar	Bipolar Monopolar	Monopolar	Monopolar
PFA/RFC	Yes/Yes	Yes/No	Yes/No	Yes/No	Yes/Yes
Dedicated 3D mapping	Yes	Yes	Yes	multiple	multiple
Approval	EU	No	No	No	EU

Several systems are currently under investigation; thus, scientific references are not always available. The assessment of clinical applicability is based on the authors’ opinion. For more details, please see text.

PFA, pulsed field ablation; PVI, pulmonary vein isolation; RF, radiofrequency; RFC, radiofrequency current.

The emendations have been made to the article.

